# Role of insulin-like growth factor-2 in Alzheimer’s disease induced memory impairment and underlying mechanisms

**DOI:** 10.3389/fncel.2024.1520253

**Published:** 2025-01-03

**Authors:** Ruiqi Chen, Xing Lu, Anqi Xiao, Junpeng Ma

**Affiliations:** ^1^Department of Neurosurgery, West China Hospital of Sichuan University, Chengdu, China; ^2^Department of Gynecological Nursing, West China Second Hospital, Sichuan University, Chengdu, China; ^3^Department of Neurosurgery, West China Tianfu Hospital of Sichuan University, Chengdu, China

**Keywords:** IGF2, memory, AD, hippocampus, treatment

## Abstract

Alzheimer’s disease (AD) is the most prevalent type of dementia. Treatments for AD do not reverse the loss of brain function; rather, they decrease the rate of cognitive deterioration. Current treatments are ineffective in part because they do not address neurotrophic mechanisms, which are believed to be critical for functional recovery. Given that structural losses are assumed to be the root cause of cognitive impairment in AD, strengthening neurotrophic pathways may be a useful preventative therapeutic approach. Insulin-like growth factor-2 (IGF2), which is widely expressed in the central nervous system (CNS), has emerged as a crucial mechanism of synaptic plasticity and learning and memory, and many studies have indicated that this neurotrophic peptide is a viable candidate for treating and preventing AD-induced cognitive decline. An increase in IGF2 levels improves memory in healthy animals and alleviates several symptoms associated with neurodegenerative disorders. These effects are primarily caused by the IGF2 receptor, which is widely expressed in neurons and controls protein trafficking, synthesis, and degradation. However, the use of IGF2 as a potential target for the development of novel pharmaceuticals to treat AD-induced memory impairment needs further investigation. We compiled recent studies on the role of IGF2 in AD-associated memory issues and summarized the current knowledge regarding IGF2 expression and function in the brain, specifically in AD-induced memory impairment.

## Introduction

**Figure 1:**
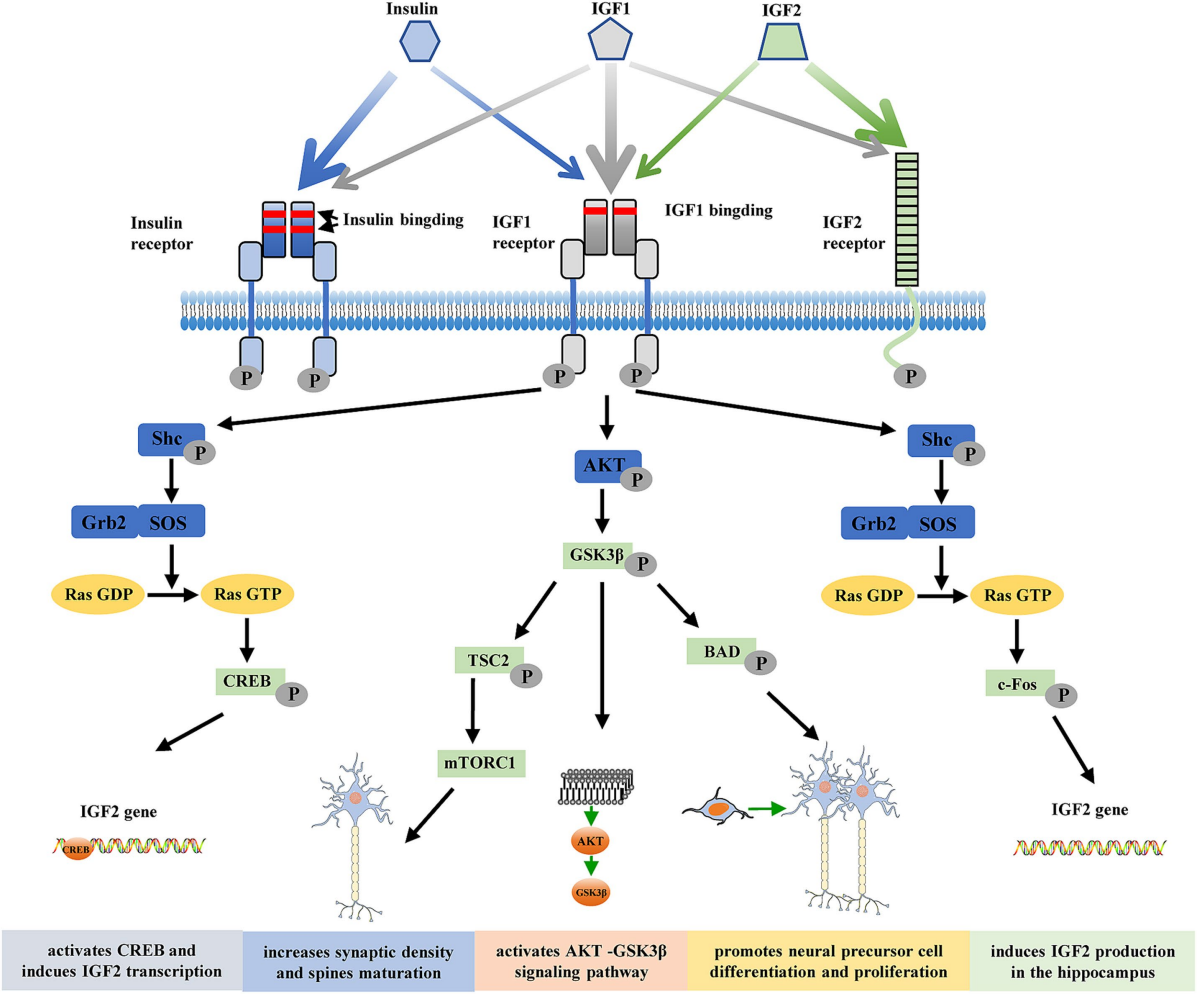
Structures of the insulin/insulin-like growth factor (IGF) system. Insulin, IGF1, and IGF2 and their relatively high-affinity receptors, insulin receptor, IGF1 receptor, and IGF2 receptor, are shown. The relative affinity of each ligand for the receptors is represented by the arrow thickness. Insulin, IGF1, and IGF2 can cross-bind to their respective high-affinity receptors; however, due to its lower affinity, insulin does not seem to bind to the IGF2 receptor. Upon receptor binding, a structural change leads to activation of the intracellular tyrosine kinase domain and autophosphorylation. Two main signaling pathways are activated: the Akt/PKB and the Ras/MAPK pathways. Thus, IGF2 activates CREB and induces IGF2 transcription, increases synaptic density and spine maturation, activates the AKT-GSK3β signaling pathway, promotes neural precursor cell differentiation and proliferation, and induces IGF2 production in the hippocampus to improve memory ability. p, phosphorylation; Shc, adaptor protein p66; Grb2, growth factor receptor-bound protein 2; SOS, son of sevenless; CREB, cAMP-response element binding; GSK3β, glycogen synthase kinase-3β; TSC2, Tuberous sclerosis complex 2; BAD, Bcl-2-associated death; mTORC1, mammalian target of rapamycin complex 1.

The pleiotropic polypeptides insulin-like growth factors 1 and 2 (IGF1 and IGF2) are found in a wide range of tissues and organs, including the central nervous system (CNS) (Wang et al., 2017). These factors are believed to mediate a wide range of physiological processes during development and adulthood and are structurally homologous with proinsulin. IGFs can function as both autocrine and paracrine factors within cells to control chemotaxis, differentiation, growth, and survival. The receptors for IGF1, IGF2, and insulin are membrane receptors that specifically mediate the biological actions of both IGFs (Dupont and LeRoith, 2001). The tyrosine kinase receptor family includes the IGF1 receptor, which shares a high degree of structural similarity with the insulin receptor. This receptor is normally found at the cell surface as a heterotetramer composed of two *α* (135 kDa) and two *β* (90 kDa) subunits linked by disulfide bonds. The receptor binds to IGF1 with greater affinity than to either IGF2 or insulin. The α-subunits have an extracellular ligand-binding site, and the *β*-subunits contain an intracellular tyrosine autophosphorylation site and transmembrane and tyrosine kinase domains (Dupont and LeRoith, 2001). Tyrosine residues in the intracellular region of the *β* subunit undergo autophosphorylation when a ligand attaches to the extracellular *α* subunit, causing a conformational shift that initiates receptor tyrosine kinase activity (Adams et al., 2000). Effector and adaptor molecules dock, activating numerous intracellular signaling cascades that control growth, proliferation, survival, development, and metabolic responses. These cascades include the phosphoinositide 3′-kinase (PI3K) and mitogen-activated protein (MAP) kinase pathways (Adams et al., 2000; Dupont and LeRoith, 2001). Unlike the insulin and IGF1 receptors, the IGF2 receptor lacks intrinsic tyrosine kinase activity and is physically different. This receptor does not bind insulin and has a greater affinity for IGF2 than for IGF1 (Figure 1) (Massagué and Czech, 1982). Although evidence that this receptor plays a role in transmembrane signal transduction in response to IGF2 binding is increasing, its biological significance is still debated.

The expression of IGF2, a neurotrophic factor, is reduced in people with Alzheimer’s disease (AD) (Pascual-Lucas et al., 2014; Xia et al., 2019). IGF2 is a modulator of hippocampal cognition that has been found to be dysregulated in numerous neurodegenerative illnesses, including AD, in recent years (Beletskiy et al., 2021; Pardo et al., 2019). Therefore, IGF2 might function as both a prophylactic therapy and traditional AD treatment (Fitzgerald et al., 2023). Here, we present increasing data suggesting that IGF2 may be a promising target for the development of novel therapies for AD and related neurological disorders.

## IGF2 and IGF2 receptor distribution in the brain

The IGF2 is produced predominantly by the choroid plexus, leptomeninges, and parenchymal vasculature in both the adult and fetal brain (Beletskiy et al., 2021; Sélénou et al., 2022). Stromal cells, in which allelic expression is inverted compared with that of peripheral tissues, also produce IGF2 in the brain. For example, most IGF2 (>90%) originates from the maternal allele in numerous rat brain areas, including the hippocampus, anterior cingulate cortex, medial prefrontal cortex, and amygdala (Ye et al., 2015). However, it is still unclear how this particular regulator works and how it functions in humans. Furthermore, it is unclear how distinct brain allelic expression is controlled in various types of brain cells under normal circumstances, whether it varies with age, and whether it changes in response to activity-dependent processes such as learning.

Additionally, the distribution of the IGF2 receptor, which is cell type specific, in the brain is unknown. Genetic knockout (KO) of the IGF2 receptor results in death at birth, highlighting the critical function of this receptor in development (Lau et al., 1994); however, little is known about the functions of the IGF2 receptor in adult tissues. The IGF2 receptor is enriched in several brain regions, including the olfactory bulb, striatum, pallidum, hypothalamus, thalamus, hippocampus, midbrain, cortex, and cerebellum, as shown by RNA hybridization and immunohistochemistry (Wang et al., 2017). IGF2 receptor expression is quite low in astrocytes, microglia, and other brain cells but is much greater in neurons at the cellular level (Yu et al., 2020). These findings suggest that IGF2 in the brain preferentially affects neuronal functions through this differential enrichment of the IGF2 receptor in neurons.

## IGF2 and AD

Currently ranked as the sixth leading cause of death globally, AD is the primary cause of dementia worldwide. In the United States, an estimated 5 million people aged 65 years and older have AD, and over the next several decades, this figure is predicted to more than double (Prince et al., 2013). The burden of AD on impacted families and the healthcare system is increasing as average life expectancy increases. Unfortunately, few effective treatments are available, even as the severity of the disease increases. According to current studies, AD is mainly associated with the aggregation of amyloid-beta (Aβ) and hyperphosphorylated tau, synaptic loss and neuronal death (Scheltens et al., 2021).

Numerous reports have connected AD with insufficient IGF2 levels. Postmortem brain tissue examination revealed that AD patient samples had significantly lower levels of IGF2 mRNA (Steen et al., 2005) and protein (Pascual-Lucas et al., 2014) in the hippocampus and hypothalamus than samples from patients without AD did. Subsequent analysis of frontal lobe samples revealed that the degree of reduction in *IGF2* mRNA was positively associated with Braak staging, a method of assessing the histology of brain tissue, and it used for assessing the progression of neuropathology. The Braak neuropathological stages are now integrated into the AD neuropathological diagnostic criteria. This histopathological classification describes the hierarchical and cumulative Tau deposition in the brain into six stages, with the following topographic hallmarks: transentorhinal cortex (Braak I); entorhinal cortex and hippocampus CA1 sector (Braak II); hippocampus (extension of damage), amygdala, and adjacent neocortical areas (Braak III); associative neocortex (initial involvement; Braak IV); associative neocortex (extension of damage), notably in temporal, parietal, and occipital areas (Braak V); primary motor and sensory fields (Braak VI). These stages coincide well with clinical manifestations: stages I–II correspond to preclinical AD, III–IV to prodromal dementia, and V–VI to fully installed de mentia. *IGF2* mRNA levels decreased to approximately 40% of those in control subjects at Braak stage 6, indicating dysregulation of IGF2 signaling in dying neurons (Rivera et al., 2005). Interestingly, IGF2 levels are increased in the cerebrospinal fluid (CSF) of AD patients, and this increase is correlated with the levels of the AD-associated CSF biomarkers phosphorylated tau and Aβ_42_ (Åberg et al., 2015; Heywood et al., 2015). IGF2 has been increasingly shown to be essential for hippocampal development and function. IGF2 may be a viable treatment option in the future since it is neurotrophic, boosts cholinergic stimulation, fosters learning and memory (Figure 1), and guards against the buildup of Aβ and its subsequent toxicity.

## Underlying mechanism by which IGF2 improves AD-induced memory impairment

### IGF2 alleviates cholinergic function impairment in AD

Cholinergics are known to influence hippocampal cognition, and one of the key pathological events in AD is a reduction in cholinergic neurotransmission (Schmeisser et al., 2012). Extracellular acetylcholine levels in the hippocampus almost double during the performance of a spatial memory task; additionally, a greater increase in acetylcholine is correlated with better task performance (Ragozzino et al., 1996). The degeneration of cholinergic neurons in the basal forebrain and decreased abundance and activity of choline acetyltransferase (ChAT), the enzyme that synthesizes acetylcholine in the presynaptic terminal, are the fundamental causes of reduced acetylcholine neurotransmission in AD patients. IGF2 has been shown to maintain cholinergic neurons and increase ChAT activity. These characteristics suggest that IGF2 might provide therapeutic benefits in the management and prevention of cholinergic dysfunction in AD patients.

For example, acetylcholine neurotransmission is dramatically increased by IGF2. IGF2 treatment caused acetylcholine release in the frontal cortex, striatum, and hippocampus in *ex vivo* rat brain slices (Kar et al., 1997). The vesicular acetylcholine transporter (vAchT) is colocalized in the hippocampus and basal forebrain, and the IGF2 receptor promotes its release (Hawkes et al., 2006). Additionally, the intrinsic responsiveness of basal forebrain cholinergic neurons is modulated by IGF2 through an IGF2 receptor-dependent mechanism. However, how the electrophysiological characteristics of these neurons influence acetylcholine neurotransmission in the cerebral cortex or hippocampus is still unknown (Hawkes et al., 2006). To determine whether exogenous IGF2 can promote hippocampal acetylcholine release *in vivo*, additional research is needed.

Furthermore, IGF2 might be able to treat cholinergic neuron degeneration, which is a part of the pathophysiology of AD. A week of chronic intracerebroventricular (ICV) IGF2 infusion increased basal forebrain cholinergic neurons while increasing hippocampal ChAT expression in a transgenic APP/PS1 animal model of AD (Mellott et al., 2014). This increase was most likely caused by increased levels of bone morphogenic factor-9 (BMP9), a neurotrophic factor that, in the same transgenic AD mouse model, has previously been shown to be a positive regulator of the health of cholinergic neurons (Burke et al., 2013). Therefore, IGF2 may both alleviate the cognitive symptoms associated with AD and partially restore the cholinergic signaling infrastructure that is frequently compromised in AD patients.

### IGF2 ameliorates hippocampal neuron synaptic function in AD

IGF2 is a strong candidate for controlling synapse formation. Dendritic spines are the main subcellular sites of excitatory synapses and are crucial for the structure and operation of the brain circuitry (Harris and Stevens, 1989). Dendritic morphology and hippocampal function, particularly learning and memory, are closely linked. A reduction in spine density, together with changes in spine architecture and associated synaptic plasticity, is correlated with decreased hippocampus-dependent memory (Martel et al., 2016; Uchida et al., 2014). According to recent research, IGF2 controls the maturation of spines and synaptic density in hippocampal neurons because of the placement of receptors in dendrites at synaptic locations. Thus, IGF2 is a novel nuclear factor kappa-B (NF-κB) neuronal target gene that regulates spine density (Schmeisser et al., 2012). In the mouse forebrain, conditional deletion of the NF-κB kinase subunit beta (IKK) NF-κB-activating enzyme was linked to decreased numbers of mature spines and postsynaptic proteins, which are critical for synaptic transmission. Furthermore, neurons lacking active IKK downregulated IGF2, and within a day, IGF2 treatment quickly increased spine formation and restored the decreased synaptic density in those neurons (Schmeisser et al., 2012). This study provides evidence that IGF2 may be an NF-κB target that regulates neuronal spine density.

Long-term potentiation (LTP), the most effective model for examining learning and memory processes at the synaptic level, can affect synaptic dendritic excitability (Bliss and Collingridge, 1993). LTP, a type of plasticity that increases the synaptic efficacy required for memory and is particularly significant for memory storage, is a quick process that can last for hours (Shors and Matzel, 1997). Stable LTP expression has been documented in the presence of IGF2 in previous studies. The benefits of IGF2 therapy on hippocampal LTP are eliminated when an IGF2 receptor antibody is used to inhibit IGF2 (Chen et al., 2011).

Phosphorylation of cAMP responsive element-binding protein (CREB), a signaling pathway that is critical for memory performance (Bourtchuladze et al., 1994), is caused by LTP induction (Schulz et al., 1999). Multiple kinases control the nuclear factor CREB, which is dormant until it is phosphorylated. CREB-dependent gene expression plays a crucial role in mediating synaptic plasticity and memory (Bartsch et al., 1998). Phosphorylated CREB levels in the hippocampus increased significantly in response to IGF2 therapy. Hippocampal injection of antisense CREB inhibited training-dependent induction, interfered with memory consolidation, and decreased training-induced expression of IGF2 (Chen et al., 2011). These findings suggested that CREB regulates IGF2, a downstream target gene (Chen et al., 2011). Recent research has demonstrated that IGF2 receptor gene expression is negatively regulated by CREB (Chen et al., 2015). However, future research should focus on understanding the relationship between CREB and IGF2 as well as the potential role that the activation of IGF2 as a downstream gene may have in mediating the effects of CREB on memory.

### IGF2 improves hippocampal neurogenesis in AD

As AD progresses, a notable indicator of neurotrophic loss is a decrease in hippocampal neurogenesis (Moreno-Jiménez et al., 2019). Age-related decreases in hippocampal neurogenesis occur in neurologically healthy individuals but are more pronounced in AD patients (Boldrini et al., 2018). Studies in rodents have suggested that hippocampal neurogenesis plays a role in numerous brain functions, including memory consolidation and neuroprotection, that are compromised in AD (Lafenêtre et al., 2010; Shors et al., 2002).

The use of adult patients who are undergoing brain irradiation to treat intracranial malignancies is crucial, although the complete relevance of adult human hippocampal neurogenesis is unknown. Neural stem cell precursors, which give rise to new neurons, are some of the non-cancerous cells that die as a result of this therapy. With symptoms ranging from mild to severe, approximately half of the patients who underwent this operation fulfilled the clinical criterion for radiation-induced cognitive decline (RICD) (Cramer et al., 2019). These findings adequately show that the loss of adult hippocampal neurogenesis is not sufficient to cause substantial cognitive impairment, although all radiation-induced cognitive decline (RICD) studies have the caveat that cognitive decline may be caused by cancer progression rather than radiation treatment. The example of these RICD patients may suggest that other disease processes (such as synaptic degeneration) have a greater influence on AD-associated cognitive decline than diminishing neurogenesis does (Duque et al., 2022; Nano and Bhaduri, 2022).

IGF2 maintains the number of neural stem cell precursors in the neurogenic zones of the hippocampus (Ziegler et al., 2014; Ziegler et al., 2012). Although IGF2 in the CSF may have an impact since these neurogenic zones are close to the lateral ventricles, these neural progenitors release IGF2 in an autocrine/paracrine manner (Ziegler et al., 2019). In a mouse model of AD, 7 days of chronic IGF2 infusion increased the expression of markers of hippocampal neurogenesis, confirming that IGF2 in the CSF can promote neural progenitors in the neurogenic zones of the hippocampus (Mellott et al., 2014). IGF2 in the CSF stimulates the growth of cortical neuron progenitors in the ventricular lining through a mechanism dependent on the IGF1 receptor (Lehtinen et al., 2011). An examination of the gene expression in hippocampal neural stem cells revealed that enhanced neurogenesis was linked to IGF2 overexpression (Bracko et al., 2012). In the same study, the proliferative action of IGF2 was reduced by an IGF1 receptor antagonist, whereas short interfering RNA (siRNA)-mediated suppression of IGF2 in the hippocampus slowed the proliferation of neural stem cell progenitors.

It may not be the best course of action for preserving cognitive capacity to choose a potential preventative treatment on the basis of increased neurogenesis because the role of adult hippocampal neurogenesis in human cognition is uncertain (Babcock et al., 2021). It is reasonable to assume that a reduction in hippocampal neurogenesis eliminates a source of new neurons, which in turn causes hippocampal atrophy. Increasing neurogenesis early in AD pathogenesis may assist in offsetting less severe structural losses, even if the extent of AD neurodegeneration suggests that lost neurons cannot be repopulated by neurogenesis.

### IGF2 decreases Aβ accumulation in AD

One pathogenic feature of AD is the accumulation of Aβ in the parenchyma, which leads to the development of amyloid plaques and high levels of soluble Aβ. There is evidence to support the idea that IGF2 may guard against neurotoxicity caused by Aβ. Endogenous IGF2 secretion was found to mediate neuroprotection against increased Aβ secretion in AD mice through an IGF1 receptor-dependent mechanism (Stein and Johnson, 2002).

In animal models of AD, IGF2 injection has been demonstrated to decrease amyloid pathology; however, the routes of administration used in these models are not appropriate for human use. Seven days of continuous IGF2 treatment decreased the number of amyloid plaques in the hippocampus of APP/PS1 mice (Mellott et al., 2014). Although soluble Aβ levels in the prefrontal and parietotemporal cortices of AD animals were decreased by adeno-associated virus (AAV)-mediated overexpression of IGF2 in the hippocampus, the hippocampus itself did not exhibit a significant decrease in plaque density (Pascual-Lucas et al., 2014). Only one intrahippocampal microinjection of IGF2 was needed to reduce the amount of soluble Aβ and the density of hippocampal plaques after 1 week (Xia et al., 2019). IGF2 has been demonstrated to decrease non-amyloid extracellular protein clumps in a mouse model of Huntington’s disease, which is consistent with its role in Aβ clearance (García-Huerta et al., 2020).

After treatment with media obtained from cultured neurons derived from AD animals, hippocampal neurons derived from wild-type mice in an *in vitro* model exhibited lower IGF2 expression, likely because of the elevated levels of Aβ in the medium (Pascual-Lucas et al., 2014). In the same study, Aβ was nearly entirely removed from the cell culture medium through viral vector-mediated overexpression of IGF2 in hippocampal neurons generated from AD mice through an IGF2 receptor-dependent mechanism. AD may include a vicious cycle of increasing pathogenic alterations and neurotrophic decline, as suggested by the observation that IGF2 attenuates Aβ-induced damage and that Aβ decreases IGF2 expression (Standridge, 2006).

One possible way that IGF2 can slow the progression of early AD is by attenuating dysregulated amyloid pathology. In fact, prophylactic treatment may not be effective unless it interferes with amyloid pathology in the presymptomatic stage. Aducanumab effectively reduced amyloid plaques but had no effect on cognitive outcomes in patients who already exhibited cognitive impairment (Howard and Liu, 2020). Studies have shown that intrahippocampal infusion of an antibody-like antagonist to oligomeric Aβ improved cognitive performance in rats fed a high-fat, high-sugar diet, supporting the theory that targeting Aβ at an earlier stage of disease might be more effective (Osborne et al., 2016). Notably, the above diet results in increased hippocampal Aβ and cognitive impairment (Osborne et al., 2016).

Despite these results, IGF2 is found in a subset of neuritic plaques containing Aβ in both AD brains and mutant APP transgenic mice (Amritraj et al., 2009; Kar et al., 2006), thus suggesting a potential role for the receptor in Aβ metabolism. In contrast to the receptor, the levels of IGF2 mRNA/peptide are reduced in AD brains (Pascual-Lucas et al., 2014; Rivera et al., 2005) and APP transgenic mice (Pascual-Lucas et al., 2014). Additionally, in experiments with two different lines of mutant APP transgenic mice, it has been demonstrated that enhancing the levels of IGF2 in the brain can ameliorate Aβ-containing neuritic plaques, synaptic deficits, and cognitive impairments (Mellott et al., 2014). Since IGF2 is capable of enhancing working memory through the IGF2 receptor (Chen et al., 2011), it is highly probable that the receptor plays a role in regulating both cognitive functions and Aβ metabolism related to AD pathology. Alternatively, considering the evidence that IGF2/M6P receptors are engaged in the intracellular trafficking of lysosomal enzymes like cathepsins B and D, which are known to regulate Aβ metabolism (El-Shewy and Luttrell, 2009; Haque et al., 2008; Nixon and Cataldo, 2006), it is likely that receptor overexpression can affect the amyloidogenic processing of APP by modifying the levels and/or redistribution of the enzymes, as is the case with cation-dependent M6P receptor overexpression (Mathews et al., 2002).

Furthermore, lysosomal enzyme leakage into the cytoplasm frequently results in cell death (Boya and Kroemer, 2008; Johansson et al., 2010). Additionally, the degradation of neurons in AD brains has been linked to chronic lysosomal activity. Given that IGF2 plays multiple roles, it is probable that unchanged receptor levels are the result of either fast turnover of the receptor or a compromise of its other roles at the expense of lysosomal enzyme delivery. As an alternative, other sorting receptors, such as the cation-dependent M6P receptor or sortilin A receptor, may be able to transport a portion of lysosomal enzymes. The cation-dependent IGF2 receptor, the levels of which are increased in susceptible neurons of the AD brain, has been shown to reroute certain lysosomal hydrolases to early endosomes and increase Aβ peptide secretion in cultured fibroblasts (Cataldo et al., 1997; Mathews et al., 2002). Therefore, more research is needed to determine the proportional importance of the IGF2 receptor in controlling lysosomal enzyme trafficking in AD pathogenesis (Table 1).

**Table 1:** Effects of IGF2 in the preclinical studies.

Animal model	Behavioral outcomes	Underlying mechanism
3 months mouse (C57Bl/6 wild-type mice)	Enhanced memory extinction	N/A (Agis-Balboa et al., 2011)
8.5 months Wistar rats	Improved memory ability	Decreased the oxidative in rats’ hippocampus and cortex (Castilla-Cortázar et al., 2011)
6 months mouse (APP.PS1/CHGFP)	Improved memory ability	Increased hippocampal neurogenesis and growth factor level in hippocampus (Mellott et al., 2014)
18 months male C57BL/6 mice and 4 and 12 months female Tg2576 mice	Alleviated the cognitive impairment	Decreased Aβ in prefrontal and parietotemporal cortices (Pascual-Lucas et al., 2014)

### Potential use of IGF2 in human treatments

IGF2 is essential for both fetal development and mammalian growth; it encourages cellular growth and survival and is a key regulator of bone formation. To create therapeutic treatments that target IGF activity in disease, a thorough understanding of how IGF2 binds with its receptors and induces downstream signaling activation is important. Currently, most methods aim to suppress IGF action by obstructing the binding of IGF1 receptor antibodies, which impedes ligand binding and promotes receptor internalization (Livingstone, 2013). For example, IGF2 inhibitors have been shown to reduce the growth of IGF2-dependent cancers. However, increases in IGF2 signaling can cause resistance to treatment (Belfiore et al., 2009; Gualberto and Pollak, 2009), emphasizing the need for IGF2 inhibitors that operate through both IGF1 receptors. These investigations will provide more insight into the specific mechanism by which IGF2 binds to the IGF1 receptor with high affinity and causes its activation. These studies will also offer recommendations for the development of inhibitors or allosteric regulators that could be used to treat diseases regulated by IGF2.

## Conclusion

IGF2 is necessary for the establishment of memory in rodents, and IGF2 treatment enhances memory. Neurodegenerative illnesses in humans are accompanied by changes in the levels of brain or circulating IGF2, indicating the critical role of IGF2 in maintaining CNS health and effective brain functions. Numerous symptoms in animal models of neurodevelopmental and neurodegenerative illnesses are improved by IGF2 treatment. The IGF2 receptor is highly abundant in neurons and is involved in lysosomal targeting, protein synthesis, and endosomal trafficking. However, little is known about the roles that IGF2 and its receptor play in the brains of different species. Subsequent research endeavors should clarify the distribution of the expression and modes of action of these proteins in neuropsychiatric disease-affected and healthy brains.

Prior research has focused mostly on IGF1 and insulin. Given the strong expression of IGF2 in the brain, further research is needed to better understand its role (Russo et al., 2005). The theory that IGF2 functions in the brain in the same way that it does in the periphery is not well supported by current data. Crucially, actions mediated by the neuronal IGF2 receptor differ from those regulated by its counterparts in peripheral and glial tissues (Zhao et al., 1999). In summary, this literature analysis offers evidence of the advantageous impact that IGF2 may have if it is injected directly into the brain to produce pro-cognitive effects. Future research should explore the possibility of long-term IGF2 therapy as a prophylactic measure for AD.
